# Benediction sign in a patient with intramedullary tumor and syringomyelia

**DOI:** 10.11604/pamj.2025.52.24.49225

**Published:** 2025-09-18

**Authors:** Nomena Finiavana Rasaholiarison, Haja Parany Rabearisoa

**Affiliations:** 1 Neurology Department, University of Fianarantsoa, Fianarantsoa, Madagascar,; 2 Neurology Department, University of Antananarivo, Antananarivo, Madagascar

**Keywords:** Benediction sign, intramedullary tumor, syringomyelia

## Image in medicine

The benediction sign is a hand gesture traditionally associated with conferring a blessing. It is a wrist position where the first three fingers are raised, which in Christian symbolism are interpreted as representing the three Persons of the Holy Trinity. A 27-year-old woman presented with an 11-month history of progressive motor deficits and sensory loss in her 4 limbs. It began with a sensory loss of the right upper and lower limbs, followed by a motor deficit in the right lower limb, left lower limb, and left upper limb. This was painless with a flexion contracture of the fourth and fifth left fingers. On physical examination, she had a benediction sign of the left hand (A), trophic skin changes, atrophy of the interosseous muscles, thenar and hypothenar eminences, spastic tetraparesia with hypopallesthesia, and sensory loss with sensory level at C5. Her spinal cord Magnetic Resonance Imaging (MRI) revealed a thickened spinal cord aspect on the T1 sequence without contrast (B), an intramedullary tumor on the T1 sequence with contrast at the C2-T1 level (C), and syringomyelia at T1-T8 (D). Unfortunately, she refused to have a biopsy of the lesion and was lost to follow-up.

**Figure 1:**
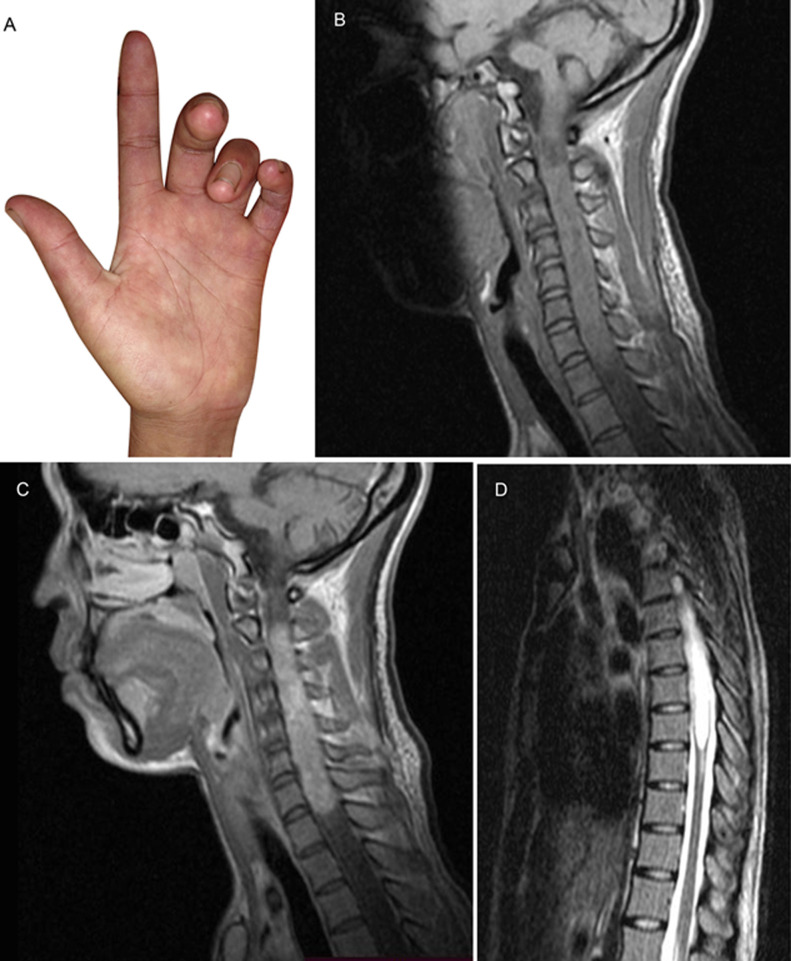
A) benediction sign, B,C) intramedullary tumor, D) syringomyelia

